# Patterns and determinants of hypertension in Botswana

**DOI:** 10.1007/s10389-015-0682-7

**Published:** 2015-07-19

**Authors:** Mpho Keetile, Kannan Navaneetham, Gobopamang Letamo

**Affiliations:** Department of Population Studies, University of Botswana, Private Bag, 00705 Gaborone, Botswana

**Keywords:** Prevalence, Chronic non-communicable diseases, Risk factors, WHO STEPS, Botswana

## Abstract

**Aim:**

This study examines the patterns and determinants of hypertension in Botswana.

**Subjects and methods:**

In 2007, a cross-sectional survey of chronic non-communicable diseases and risk factors was conducted by the Ministry of Health and World Health Organisation using the STEPS approach. STEP 1 was the collection of demographic data, STEP 2 was the physical measurement of the height, weight, waist and hips, and blood pressure; STEP 3 was biochemical measurements, which included the collection of blood samples. A nationally representative sample of 4003 individuals aged 25–64 years was included for analysis.

**Results:**

From a total sample of 4003 respondents, the national prevalence of hypertension was estimated to be 16.9 % (9.9 % for males versus 18.9 % for females). Logistic regression analysis indicated a positive association between gender and hypertension prevalence, with females (OR 1.9) more likely to be hypertensive. Hypertension increases significantly with age for both males and females. For women a high educational level and employment status were not associated with hypertension. Meanwhile, of all the behavioural risk factors, obesity was the only one with a significant association with hypertension.

**Conclusion:**

The implications of this study are that a reduction in obesity through a balanced diet and increased physical activity will have far-reaching results in lowering hypertension. Botswana’s health system should place greater emphasis on the detection of hypertension at early ages and create awareness programmes for both the general population and health personnel with respect to the detection, treatment and control of hypertension.

## Introduction

Hypertension (commonly known as high blood pressure) is increasingly becoming an important medical and public health issue worldwide. It is estimated that high blood pressure has caused an estimated 7.6 million premature deaths (13.5 % of the total), and it contributed to 92 million disability-adjusted years (DALYs) worldwide in 2001 (Lopez et al. 2006). Modelled projections indicate an increase to 1.15 billion hypertensive patients by 2025 in developing countries, which is an increase of 80 % as compared with hypertensive patients in 2000 (Lopez et al. 2006). When looking at sub-Saharan Africa, it has been suggested that the prevalence of cardiovascular disease and hypertension is increasing rapidly (Seedat 2004). Some studies have shown that the current prevalences of hypertension in many developing countries, particularly in urban societies, is already as high as those seen in developed countries (Boutayeb 2006 and Vorster 2002). Recent epidemiological studies in sub-Saharan Africa have shown that communicable diseases such as malaria and HIV are still the major causes of morbidity and mortality (Omeleke 2013), contributing to a dual burden for health care provision and a threat to population health.

Previous research on hypertension in Africa has shown that many studies provide data on hypertension in sub-Saharan Africa (World Health Organization 2011; Young et al. 2009; Abengunde et al. 2007; Chobanian et al. 2003), but very few of these provide detailed analysis of the patterns and determinants of hypertension. These studies have generally shown a high prevalence of hypertension among urban populations and also that hypertension increases with age (Abengunde et al. 2007; Chobanian et al. 2003). Regarding the epidemiological profile of Botswana, HIV/AIDS has been the leading cause of morbidity and mortality since it was first discovered in 1985 (Ministry of Health 2008). The burden of HIV/AIDS and other infectious diseases has led the government of Botswana to neglect chronic non-communicable diseases even though they have been increasing in recent years.

Trends in ill health in Botswana have shown that there has been a decline in childhood infectious diseases and an increase in chronic non-communicable diseases and their associated risk factors. For instance, the Ministry of Health and WHO (Ministry of Health 2008) found an increase in non-communicable diseases, especially hypertension, cancer and diabetes, in Botswana, and analysis from the health statistics reports shows that the past 18 years have seen an unprecedented increase in hypertension prevalence (Central Statistics Office 2008). Hypertension was observed to be a more common cause of morbidity and mortality related to cardiovascular diseases and has increased five-fold over the 1980–1998 period (Central Statistics Office 2008), and it has continued to increase over the years (Central Statistics Office 2008). Risk factors underlying hypertension prevalence are well documented in the literature. They include tobacco and alcohol consumption, an unhealthy diet (low in fruits and vegetables and high in salt, fat and sugar), physical inactivity (sedentary lifestyle), raised blood pressure and obesity (BP) (Mufunda et al. 2000).

The burden of hypertension has been seen to be unequally distributed among different social classes, and its risk factors also show variations between males and females (Wong et al. 2014). Furthermore, the prevalence of hypertension has also been observed to vary with respect to various behavioural factors such as lack of physical activity, smoking and alcohol consumption. In developing countries obesity has been positively associated with high socio-economic status leading to high risk factors for non-communicable diseases (NCDs), in particular hypertension and diabetes (Wong et al. 2014). Despite the evidence that hypertension is one of the most common non-communicable diseases in Botswana, there is little evidence of research on this risk factor for cardiovascular disease. This article is an attempt to assess the patterns of prevalence of hypertension and to identify its determinants with respect to socio-economic and behavioural factors. Further, an attempt has been made to identify how the determinants of hypertension vary by gender. The results of this analysis will have important policy implications for identifying potential population groups at risk and designing targeted cost-effective interventions at both the population and individual level in order to reduce the burden of hypertension, which is a major risk factor for coronary artery disease, hypertensive heart disease, haemorrhage and stroke, and hypertensive kidney failure.

## Methods

### Data source and sampling design^1^

The article uses secondary data derived from the 2007 WHO STEPS survey,^2^ which was a population-based survey of adults aged 25–64 years. The 2001 population and housing sampling frame was adopted, and a multi-stage sampling procedure was used for the 2007 STEPS survey. The survey included 4003 individuals aged 25–64 years, covering both rural and urban areas. The survey was carried out in three steps, where STEP 1 was the collection of demographic and behavioural aspects; STEP 2 was the physical measurement of the height, weight, waist and hips; blood pressure and pulse rate: STEP 3 was biochemical measurements, including the collection of blood samples. The WHO STEPS survey has been conducted in many developing countries, including Botswana, for the surveillance of non-communicable diseases using validated instruments. For details on the sampling design and data collection method, refer to (Ministry of Health 2010).

### Measurement of variables

#### Dependent variables

This study used the *prevalence of hypertension* as the dependent variable, and it was measured by the following questionnaire items: “in the past 12 months have you been told by a doctor or other health worker that you have raised blood pressure (BP) or hypertension? The binary dependent variable was created such that if the response was ‘yes’ it was coded as 1 and 0 otherwise”. Some studies have used this question to measure hypertension prevalence (see, Jing et al. 2013; Johnston et al. 2007) because it is only at a severely elevated level of BP that an individual may be told by medical practitioners that they have hypertension or raised blood pressure (Wong et al. 2014).^3^

#### Explanatory variables

The major risk factors considered to be associated with hypertension are daily smoking, alcohol consumption, poor fruit and vegetable consumption, lack of physical activity and obesity. Confounding variables such as age, education and employment status were used as controls to investigate the association between risk factors and hypertension. The list of socio-economic and behavioural variables and their measurement is given in Table 1.

**Table 1:** Measurement of socio-economic and behavioural factors

Behavioural factors	Measure/question
Current daily smoking	Do you currently smoke tobacco products daily? Yes is coded 1 and no 0
Hazardous drinking/alcohol consumption	During the past 7 days, how many standard drinks of any alcohol did you have each day. Hazardous drinking is defined as 40–59.9 g (4–<6 drinks) of pure alcohol on average per day for men and 20–39.9 g (≥2–<4 drinks) for women. A standard drink contains approximately 10 g of pure alcohol. Three or more drinks per day for both genders has been coded 1 to denote hazardous drinking and 0 otherwise
Lack of physical activity	Do you do any activity that involves moderate intensity sports, fitness or recreational activities that cause large increases in breathing or heart rate for at least 10 min? Yes is coded 1 and no 0
Poor vegetable consumption	In a typical week, how many days do you eat vegetables? If individuals reported that they did not eat any vegetables on any 1 of the 7 days of a week, then this poor vegetable consumption was given code 1 and 0 otherwise
Obesity	Calculated from the body mass index (BMI), defined as the weight in kilograms divided by the square of the height in metres (kg/m2). The following BMI values were used: <18.50 = underweight; 18.50–24.99 = normal weight; 25–29.99 = overweight; ≤30 obese. Code 1 is given to overweight and obesity while 0 is for underweight and normal weight
Socio-economic factors	(i)Sex: male coded 1, female 0 (ii)Age: 25–34 coded 1, 35–44 = 2, 45–54 = 3 and 55–64 = 4 (iii)Education: For education, the following categories were created: (i) primary or less =1 (derived by combining non-formal education, less than primary and primary school completed); (ii) secondary =2 (derived from secondary school completed, junior secondary school completed, senior secondary school completed, high school completed); (iii) tertiary or higher = 3 (derived from tertiary school completed, college/university completed, postgraduate degree) (iv)Type of employment = government employee coded = 1, non-government employee = 2, self-employed = 3 and unpaid worker = 4

### Data analysis

Binary logistic regression was used to identify the major determinants of hypertension. Logistic regression results are presented in the form of adjusted odds ratios, which explain the probability of having hypertension in a particular category of variable in comparison with the reference category while controlling other factors. Data for this article were analysed using the Statistical Package for Social Sciences (SPSS) programme, version 21. Three models were used. Model I presents the probability of having hypertension by risk factor variables for the whole population (sample), while controlling for socio-economic status. Model II presents the probability of having hypertension by risk factor variables for men, controlling for their socio-economic status, while model III measures the probability of having hypertension by risk factors for women, controlling for their socio-economic status. Logistic regression results are presented as odds ratios together with their 90 % and 95 % confidence intervals.

## Results

### Sample description

Table 2 presents characteristics of the study population by gender. Results indicate that male respondents constitute about one third (32.1 %) of the sample, while females accounted for the remaining two thirds (67.9 %). Among both males and females the majority of them belong to the 25–44 age group. About 40 % of males and 52 % of females have primary school education or less. A small proportion of females (13.4 %) had tertiary education compared to males (23.3 %). Male respondents who were doing unpaid work (36.4 %) constituted a large proportion when considering employment status, followed by government employees (28.4 %), non-government employees (22 %) and self-employed (13.2 %) males. Among female respondents, more than half (55.2 %) were doing unpaid work, followed by non-government employees (22 %), government employees (12.2) and lastly self-employed (10.3 %).

**Table 2:** Distribution of the study population by demographic, socio-economic and behavioural factors for males and females

Variable	Males (*n* = 1284, 32.1 %)		Females (*n* = 2719, 67.9 %)	
	N-	%	N-	%
Age				
25–34	537	41.8	919	33.8
35–44	320	24.9	666	24.5
45–54	182	14.2	623	22.9
55–64	245	19.1	511	18.8
Education				
Primary or less	517	40.3	1426	52.4
Secondary	468	36.4	928	34.1
Tertiary or higher	299	23.3	365	13.4
Type of employment				
Government employee	365	28.4	339	12.4
Non-government employees	282	22.0	599	22.0
Self-employed	169	13.2	280	10.3
Unpaid workers	467	36.4	1501	55.2
Do you currently smoke any tobacco products such as cigarettes, cigars?				
Yes	425	33.1	239	8.8
No	859	66.9	2480	91.2
Hazardous drinking				
Yes^a^	660	48.4	464	17.1
No	664	51.6	2255	82.9
Do you do any moderate-intensity sports, physical fitness or recreational activities that cause small increases in breathing?				
Yes	344	26.8	125	4.6
No	940	73.2	2594	95.4
In a typical week on how many days do you eat vegetables?				
None	126	9.7	245	9.0
One/more days	1158	90.3	2474	91.0
Obesity				
Yes	78	6.0	660	24.1
No	1206	94	2059	75.9

^a^All alcohol consumers were hazardous drinkers for both males and females

The risk factors varied greatly between males and females. The results indicate that among males, one third (33.1 %) of the respondents reported that they were currently smoking tobacco products, while among females under a tenth (8.8 %) reported that they were currently smoking. Furthermore, the results show that slightly under half (48.4 %) of the male respondents were hazardous drinkers, while slightly over a sixth (17.1 %) of the female respondents were hazardous drinkers. More than seven out of ten (73.2 %) male respondents indicated that they do not do any moderate-intensity sports, physical fitness or recreational activities that cause small increases in breathing, while among females the proportion of respondents who do not do physical exercise was relatively higher, accounting for 95.4 %.

The proportion of respondents who do not eat vegetables on any day of the weeks was almost the same among males (9.7 %) and females (9 %). Furthermore, the results indicate that obesity is relatively higher among female than male respondents; for instance, only 6 % of the male respondents were obese, while 24.1 % of the female respondents were reported to be obese.

### Patterns of hypertension prevalence

Table 3, presents the results concerning the prevalence of hypertension for each category of independent variable, stratified by gender. Hypertension has been observed to be more prevalent among females (18.9 %) than males (9.9 %). The results also indicate the prevalence of hypertension increases with age for both genders, although the prevalence is high among female respondents. For instance, among males, the hypertension prevalence in the ages 25–34, 35–44, 45–54 and 55–64 years was 5.3, 6.7, 11.8 and 18.5 % respectively, while among females, the prevalence in ages 25–34, 35–44, 45–54 and 55–64 years was 6.6, 17.3, 28.4 and 41.6 % respectively. For all ages, the prevalence of hypertension is higher among female respondents.

**Table 3:** Prevalence of hypertension by demographic, socio-economic and behavioural factors among males and females

Variable	Male (*n* = 1284)		Female (*n* =2719)	
	%	N-	N-	%
Age				
25–34	5.3	537	6.6	919
35–44	6.7	320	17.3	666
45–54	11.8	182	28.4	623
55–64	18.5	245	41.6	511
Education				
Primary school or less	11.2	517	23.9	1426
Secondary	6.8	468	12.1	928
Tertiary or higher	12.6	299	16.7	365
Type of employment				
Government employee	13.2	365	20.8	339
Non-government	8.8	282	19.3	599
Self-employed	10.1	169	20.2	280
Unpaid workers	7.6	467	17.5	1501
Do you currently smoke any tobacco products such as cigarettes, cigars or pipes?				
Yes	7.9	425	20.7	239
No	10.9	859	18.7	2480
Hazardous drinking				
Yes	10.2	664	18.8	2255
No	9.5	660	19.6	464
Do you do any moderate-intensity sports, physical fitness or recreational activities that cause small increases in breathing?				
Yes	7.1	344	15.3	125
No	11.0	940	19.0	2594
In a typical week, how many days do you eat vegetables?				
None	9.4	126	21.8	245
One/more days	9.9	1158	18.6	2474
Obesity				
Yes	31.1	78	29.1	660
No	8.6	1206	15.6	2059

Among male respondents the prevalence of hypertension was significantly high among respondents with tertiary or higher education (12.6 %), followed by those with primary school or less (11.2 %) and secondary education (6.8 %). Interestingly, among females the prevalence of hypertension was significantly high with primary school education or less (23.9 %) compared to respondents with tertiary (16.7 %) and secondary education (12.1 %). Furthermore, among male respondents hypertension was more prevalent among those who were government employees (13.2 %) than among self-employed, non-government employees and unpaid workers. Meanwhile, among female respondents, the prevalence of hypertension was more pronounced among government employees (20.9 %) and the self-employed (20.2 %) than among non-government employees (19.3 %) and unpaid workers (17.5 %).

The prevalence of hypertension among current smokers was significantly high among females (20.7 %) compared to males (7.9 %). The results also indicate that the proportion of respondents with hypertension was marginally high among both males and females who consume alcohol than those not consuming alcohol. The results also indicate that males who reported that they were not physically active had a higher propensity to have hypertension (11 %) than those who were physically active (7.1 %). A similar finding was also observed for females.

The study did not find any significant difference between those who consume vegetables frequently and those who do not in the case of males. On the other hand, for females, the proportion at risk for hypertension was high among those who had not eaten vegetables (21.8 %) on any 1 day of the week compared to those who ate vegetables on 1 or more days of the week (18.6 %). The study found that obesity seems to be an important covariate for hypertension. For males, hypertension was more prevalent among those who were obese (31.1 %) than those who were not (8.6 %), and also for females hypertension was more pronounced among those who were obese (29.1 %) than those were not (15.6 %). The results also show that the risk of hypertension is greater among obese men (31.1 %) than obese women (29.1 %).

### Determinants of hypertension

Table 4 shows the adjusted odd ratios estimated from the logistic regression models by including the demographic, socio-economic and risk factors as covariates for hypertension. The binomial dependent variable was a respondent’s reported status of hypertension (yes = 1; no = 0). The results included the three models separately for males, females and the total.

**Table 4:** Adjusted odds ratios (OR) and 95 % confidence intervals (CIs) for the probability of having hypertension for the total, males and females

Variable	Model I (total)	Model II (males)	Model III (females)
	(*N* = 3556)	(*N* = 1143)	(*N* = 2407)
Risk factors			
Daily smoking			
Yes	0.85 (0.629–1.137)	0.83(0.552–1.248)	0.92(0.593–1.433)
No	1.0	1.0	1.0
Hazardous drinking			
Yes	1.17(0.920–1.497)	1.13(0.791–1.631)	1.20(0.863–1.677)
No	1.0	1.0	1.0
Lack of physical activity			
Yes	1.07(0.744–1.537)	1.33(0.838–2.099)	0.89(0.469–1.725)
No	1.0	1.0	1.0
Poor vegetable consumption			
Yes	1.11(0.798–1.554)	1.05(0.590–1.870)	1.14(0.753–1.727)
No	1.0	1.0	1.0
Obesity			
Yes	2.12*(1.679–2.670)	3.81*(2.370–6.134)	1.74*(1.337–2.267)
No	1.0	1.0	1.0
Explanatory variables			
Education			
Primary or less	1.0	1.0	1.0
Secondary	1.00(0.765–1.310)	1.09(0.688–1.750)	1.02(0.730–1.425)
Tertiary/higher	1.29(0.946–1.776)	1.67*(1.028–2.703)	0.95(0.607–1.489)
Type of employment			
Government employees	1.48*(1.104–1.986)	1.93*(1.227–3.044)	1.28(0.842–1.939)
Non-government employees	1.25** (0.967–1.623)	1.60** (0.982–2.608)	1.13(0.827–1.534)
Self-employed	1.12(0.817–1.541)	1.31(0.764–2.246)	1.04(0.697–1557)
Unpaid workers 1.0	1.0	1.0	1.0
Control variables			
Age			
25–34	1.0	1.0	1.0
35–44	2.10*(1.568–2.816)	1.48*(0.920–2.382)	2.57*(1.759–3.744)
45–54	3.73*(2.478–5.074)	2.88*(1.729–4.779)	4.47*(3.015–6.619)
55–64	7.05*(5.038–9.861)	6.33*(3.602–11.104)	7.89*(5.145–12.101)
Sex			
Male	1.0		
Female	1.92*(1.518–2.422)		

*Statistically significant at *P* < 0.05; **statistically significant at *P* < 0.1

The results of model I indicate females have a greater risk of hypertension than males. For instance, the probability of having hypertension among females was 1.9 times higher than among males when controlling for age and other socio-economic and risk factors. It was also shown that the probability of having hypertension increases with age; for instance respondents aged 35–44 (OR, 2.101), 45–54 (OR, 3.734) and 55–64 years (OR, 7.048) were 2, 3 and 7 times more likely to have hypertension respectively compared to those aged 25–34 years. Furthermore, the results indicate that hypertension was more likely to be prevalent among government employees (OR, 1.481) and non-government employees (OR, 1.253) than among unpaid workers after controlling for other covariates. The results also indicated that the risk factors daily smoking, alcohol consumption, lack of physical activity and poor vegetable consumption were not significantly related to the probability of having hypertension. The only behavioural risk factor that has a greater effect on the hypertension was obesity. For instance, the results indicate that the respondents who were obese were two times (OR, 2.118) more likely to have hypertension compared to those who were not obese.

The results of model II indicate that the odds of having hypertension increase with age for males; for instance, men aged 35–44, 45–54 and 55–64 years were 1.4, 2.8 and 6.3 times more likely respectively to have hypertension compared to men aged 25–34 years. Men who had a tertiary education (OR = 1.667) were more likely to have hypertension compared to those with primary education or less, while government employees (OR = 1.932) and non-government employees (OR = 1.600) were also more likely to have hypertension compared to unpaid workers. Behavioural risk factors such as daily smoking, alcohol consumption, lack of physical activity and poor vegetable consumption were not found to have a significant association with the risk of having hypertension. However, obesity was found to be an important determinant of hypertension among males. Men with obesity have 3.8 times higher probability of having hypertension compared to their non-obese counterparts.

Model III gives the adjusted odd ratios for logistic analysis for females. As observed in the case of males, as age increases, among females, the odds of having hypertension also increased. For instance, females aged 35–44 (OR = 2.566), 45–54 (OR = 4.467) and 55–64 (OR = 7.89) years were more likely to have hypertension compared to young women aged 25–34 years. Surprisingly, in the case of females, the level of education and employment status were not significantly associated with hypertension. Analogous to males, the odds of the behavioural risk factors such as daily smoking, alcohol consumption, lack of physical activity and poor vegetable consumption on hypertension were not significant. As expected, women who were obese were 1.7 times (OR, 1.741) more likely to have hypertension than their non-obese counterparts.

## Discussion

The emergence of cardiovascular diseases in Botswana during the past 2 to 3 decades has inspired the policy makers to devise a policy to combat these diseases. The Ministry of Health in Botswana and World Health Organisation have made efforts toward the surveillance of NCDs and their risk factors in Botswana. This study used the WHO STEPS 2007 data to understand the patterns and major determinants of hypertension in Botswana. This is the first study in the context of Botswana to analyse the major risk factors for hypertension, which is a major cause of cardiovascular disease.

The study noted that a gender differential in the prevalence of hypertension is dominant in Botswana. The propensity among females to have hypertension was twice that of males. This was also confirmed after adjusting for age and other covariates in the logistic regression model. As expected, age has a significant positive association with the probability of having hypertension for both males and females. This is in conformity with some other studies on the prevalence of hypertension that found that hypertension also increases with age, and this is the case for both developed and developing countries (Prince et al. 2012; World Health Organization 2010; Mohan et al. 2005).

The level of education and employment status were significant only for males but not for females. Similar findings were observed in Vietnam: women with low socio-economic status were more often hypertensive than those with high occupational and educational statuses (Son et al. 2012). In other developing countries an inverse association between hypertension and higher education was found for all countries except India (Brummett et al. 2011), where education had no effect. Meanwhile, in Ghana, only people with no education were less likely to have hypertension (Bosu 2010). Furthermore, a recent study by Cois and Erhlich (2014) in South Africa found that among women high education and income levels were associated with a drop in systolic and diastolic blood pressure. Meanwhile for men, in contrast, an increase in both education and income was associated with an increase in blood pressure levels (Cois and Erhlick 2014), as also observed in the current study. Men with tertiary education and working in either government or non-government organisations have greater risk for having hypertension than their counterparts. It is supposed that men who are not working in government and non-government organisations are doing manual labour type of works, making them at less risk of hypertension.

Unlike other previous studies in sub-Saharan Africa (Gebreselassie and Padyab 2014; Hendriks et al. 2012), the risk factors such as smoking, alcohol consumption, physical inactivity and poor vegetable consumption were not found to have a significant effect on the odds of hypertension for either males or females. The study found that obesity seems to be an important covariate for hypertension in Botswana. This finding was pervasive across genders. Also the effect of obesity on hypertension among males was twice that of females. Generally, the literature on hypertension identifies obesity as one of the major determinants of hypertension (Vernooij et al. 2012; Maniecka-Bryla et al. 2011; Commodore-Mensah et al. 2014; Abubakari and Bhopal 2008). Furthermore, some studies have noted that obesity is a major risk factor for conditions such as diabetes, hypertension, dyslipidemia, coronary heart disease and reduction of life expectancy (Son et al. 2012; Mohan et al. 2005).

The findings of this analysis reconfirmed the earlier findings that obesity is strongly associated with increased levels of hypertension. The findings of this article perhaps indicate a confirmation of this notion because women were observed to be hypertensive more often than men, and this could be associated with the ripple effect of obesity on hypertension. The association between obesity and hypertension in sub-Saharan Africa is well documented in many countries, for instance, Eritrea. Mufunda et al. (2006) found this strong association, as did Commodore-Mensah et al. (2014) in West African countries. The study also noted that the prevalence of obesity was high among females. A similar finding was also observed in urban Tanzania where women had a 4.3 times of odds of obesity compared to men (Njelekela et al. 2009), and it has been observed that being obese predisposes women to high risks of hypertension more than men (Marie et al. 2014). A study on the global, regional and national prevalence of overweight and obesity in children and adults during 1980–2013 by Marie et al. (2014) also reported that obesity was higher among women than men, and this continued over time in developing countries.

## Conclusion

Botswana is undergoing a major health transition and suffering from the dual disease burden of HIV/AIDS and non-communicable diseases. The implication of this study is that reducing obesity by a balanced diet and increased physical activity will have far-reaching results in lowering hypertension and in turn reducing cardiovascular disease in Botswana. Botswana’s health system should place greater emphasis on the detection of hypertension at early ages and create awareness programmes for both the general population and health personnel with respect to the detection, treatment and control of hypertension.

## Footnotes

1 The sampling design was adopted from the STEPS Survey Report (2010).

2 WHO STEPS Survey: These are surveys that prescribe three steps—a questionnaire, physical measurements and biochemical measurements. The steps consist of core items, core variables and optional modules. Core topics covered by most surveys are the demographics, health status and health behaviours. These provide data on socio-economic risk factors and metabolic, nutritional and lifestyle risk factors.

3 Note that if individuals are aware that they have hypertension because they were told by medical practitioners but we do not consider it a chronic illness, our measure will underestimate the individual’s knowledge of their hypertensive status. Furthermore, most health surveys use this type of question as indicated above to measure self-reported hypertension prevalence.
